# The Effect of Acute Intermittent and Continuous Hypoxia on Plasma Circulating ßOHB Levels Under Different Feeding Statuses in Humans

**DOI:** 10.3389/fphys.2022.937127

**Published:** 2022-07-06

**Authors:** Caroline Marcoux, Renée Morin, Jean-François Mauger, Pascal Imbeault

**Affiliations:** ^1^ School of Human Kinetics, Faculty of Health Sciences, University of Ottawa, Ottawa, ON, Canada; ^2^ Institut du Savoir Montfort, Hôpital Montfort, Ottawa, ON, Canada

**Keywords:** ß-hydroxybutyrate, ketone bodies, fatty acids, hypoxia, adipose tissue insulin resistance index (adipo-IR)

## Abstract

**Introduction:** Acute hypoxia is known to increase circulating nonesterified fatty acid (NEFA) levels. Adipose tissue lipolysis is a major source of NEFA into circulation and insulin suppresses this process when the tissue is insulin sensitive. NEFA can be esterified to triglycerides and/or completely/partially oxidized, the latter leading to ketogenesis in the liver. To our knowledge, the effect of hypoxia on ketogenesis, more specifically ß-hydroxybutyrate (ßOHB) levels, remains unknown in humans. Therefore, the objective of this study was to determine the effect of acute intermittent and continuous hypoxia on circulating ßOHB levels under different feeding status.

**Methods:** Plasma samples from three different randomized crossover studies were assessed for ßOHB concentrations. In the first study, 14 healthy men (23 ± 3.5 years) were exposed to 6 h of normoxia or intermittent hypoxia (IH-Fed) (15 hypoxic events/hour) following an isocaloric meal. In the second study, 10 healthy men (26 ± 5.6 years) were exposed to 6 h of continuous normobaric hypoxia (CH-Fasted) (FiO_2_ = 0.12) or normoxia in the fasting state. In the third study (CH-Fed), 9 healthy men (24 ± 4.5 years) were exposed to 6 h of normoxia or CH in a constant prandial state. ßOHB, NEFA and insulin levels were measured during all sessions.

**Results:** In the IH-Fed study, ßOHB and NEFA levels tended to be greater over 6 h of IH (condition × time interaction, ßOHB *p* = 0.108 and NEFA *p* = 0.062) compared to normoxia. In the CH-Fasted study, ßOHB and NEFA levels increased over time in both experimental conditions, this effect being greater under CH (condition × time interaction, ßOHB *p* = 0.070; NEFA *p* = 0.046). In the CH-Fed study, ßOHB levels slightly increased up to 180 min before falling back to initial concentrations by the end of the protocol in both normoxia and CH (main effect of time, *p* = 0.062), while NEFA were significantly higher under CH (*p* = 0.006).

**Conclusion:** Acute normobaric hypoxia exposure tends to increase plasma ßOHB concentrations over time in healthy men. The stimulating effect of hypoxia on plasma ßOHB levels is however attenuated during postprandial and prandial states.

## Introduction

Ketone bodies (KB), namely acetoacetate (AcAc) and ß-hydroxybutyrate (ßOHB) (Robinson and Williamson, 1980), are 4 carbons, organic molecules, commonly recognized as a substrate. In fed and short-term fasted resting healthy humans, plasma levels of ßOHB are about twice those of AcAc (Newman and Verdin, 2014a), making ßOHB a tracer of choice for assessing ketogenesis (Balasse and Féry, 1989; Veech, 2004). Both KB are formed mainly in the liver from the partial oxidation of fatty acids (FA) into acetyl-CoA. The most well-known purpose of KB is to serve as an alternative oxidative substrate for the brain in situations of decreased glucose availability (Owen et al., 1967; Balasse and Féry, 1989), as during fasting or very low carbohydrate diets (McDonald and Cervenka, 2018). However, recent observations also attributed regulatory functions to KB (Shimazu et al., 2013; Newman and Verdin, 2014b), such as antilipolytic properties (Taggart et al., 2005) and a propensity to modulate the sympathetic nervous system activity (Kimura et al., 2011; Won et al., 2013). It is often underrated that KB are continuously produced (Balasse and Féry, 1989). In healthy individuals, ketonemia follows a circadian cycle with a peak around midnight and a nadir in the morning (Wildenhoff, 1975; Robinson and Williamson, 1980).

KB kinetics are mainly impacted by plasma nonesterified fatty acids (NEFA) concentrations and circulating hormones (particularly insulin, glucagon and catecholamines) (McGarry and Foster, 1977; Keller et al., 1989; Laffel, 1999; Cahill, 2006; Puchalska and Crawford, 2017). Circulating NEFA are mostly derived from the breakdown of triglycerides (TG) in adipose tissues (Farkas et al., 1973), which is governed by the action of different hormones such as insulin, glucagon and catecholamines (Barrows and Parks, 2006). Consequently, increased ketonemia is observed both in a fasted state which lowers insulinemia, and in states of increased metabolic need, for instance during exercise or stress (Balasse et al., 1978; Féry and Balasse, 1983; Balasse and Féry, 1989), which increases catecholamines and glucagon secretion. Under such circumstances, the increased delivery of NEFA to the liver stimulates their oxidation through β-oxidation, which produces great amounts of acetyl-CoA that feeds ketogenesis. Conversely, the fed state is generally associated with reduced ketonemia (Walsh et al., 2013; Geisler et al., 2016). The typical postprandial increase in insulinemia both inhibits adipose tissue lipolysis (Jensen et al., 1989; Laffel, 1999), which reduces the delivery of NEFA to the liver, and inhibits the transport of long chain FA into the mitochondria (Laffel, 1999) which suppresses fatty acid oxidation. Hence, the channeling of liver lipids toward re-esterification (for storage and secretion as very-low density lipoproteins) should prevent β-oxidation and ketogenesis. It should however be noted that significant increase in plasma KB levels were reported in men and women following oral fat loading tests (Meijssen et al., 2000; Halkes et al., 2003).

Plasma NEFA fluctuates according to several physiological conditions, notably an activation of the sympathetic nervous system, as observed upon hypoxia exposure (Somers et al., 1989; Eichhorn et al., 2017). Hypoxia is a state of oxygen deficiency (Sjöberg and Singer, 2013) that can manifest intermittently such as during obstructive sleep apnea (Drager et al., 2010) or continuously such as during high altitude exposure or lung diseases (Vanier et al., 1963). Under hypoxic conditions, oxygen availability becomes limited and oxidative phosphorylation is hampered (Solaini et al., 2010). Experiments in humans studying the metabolic responses to hypoxia in continuous and intermittent forms, and under fed or fasted state, showed that hypoxia significantly increases plasma NEFA concentrations (Jun et al., 2011; Mahat et al., 2016, 2018; Chopra et al., 2017; Mauger et al., 2019; Morin et al., 2021), which should translate into an increased ketogenesis. In this regard, limited studies conducted in rodents have reported that circulating ßOHB levels significantly increase in response to acute hypoxic exposure (D’Alecy et al., 1990; Jun et al., 2012; Rising and D’Alecy, 1989). Nevertheless, to our knowledge, it remains undetermined whether hypoxia increases ketonemia in humans.

Thus, to examine the effect of hypoxia on circulating levels of KB in humans, we measured ßOHB concentrations in plasma samples from three previous studies. The first study assessed healthy individuals acutely subjected to normobaric intermittent hypoxia following a high fat liquid meal (referred to as IH-Fed) (Mahat et al., 2016). In the second study, healthy individuals were acutely exposed to continuous normobaric hypoxia in the fasted state (referred to as CH-Fasted) (Mahat et al., 2018). In the third study, healthy individuals were subjected to continuous hypoxia in a constantly fed state [referred to as Continuous Hypoxia-Fed (CH-Fed)] (Mauger et al., 2019). We retrospectively analyzed those studies to assess the acute effects of two main forms of hypoxia, intermittent and continuous, under different feeding statuses, on ketonemia. We hypothesized that the rise in circulating NEFA levels under acute normobaric hypoxia would increase plasma ßOHB concentrations.

## Materials and Methods

### Participants

Healthy male volunteers (*n* = 14, 23 ± 3.5 years, for IH-Fed study; *n* = 10, 26 ± 5.6 years, for CH-Fasted study; *n* = 9, 24 ± 4.5 years, for CH-Fed) were recruited for the study. Participant characteristics are summarized in Table 1. The health status of our participants was defined as an absence of current or past diagnosis for metabolic, cardiac, or respiratory issues through a medical questionnaire. The participants also demonstrated resting heart rate, blood pressure (Rosendorff et al., 2015) (data not shown), glucose (Szablewski, 2020), ßOHB (Robinson and Williamson, 1980) and fasting HOMA-IR (Matthews et al., 1985) levels that are within normal ranges (Table 2).

**TABLE 1:** Characteristics of participants in the intermittent hypoxia (IH-Fed) and continuous hypoxia (CH-Fasted and CH-Fed) studies.

	IH-fed	CH-fasted	CH-fed
Participants	*n* = 14	*n* = 10	*n* = 9
Age (y)	23 ± 3.5^a^	26 ± 5.6^a^	24 ± 4.5^a^
Height (cm)	180.5 ± 6.6^a^	177.9 ± 4.7^a^	178.9 ± 3.6^a^
Weight (kg)	85.5 ± 11.8^a^	79.9 ± 8.9^a^	77.8 ± 8.0^a^
Body mass index (kg/m^2^)	26.2 ± 3.5^a^	25.2 ± 2.5^a^	24.3 ± 2.6^a^
Lean mass (kg)	66.2 ± 8.3^a^	58.6 ± 6.7^b^	65.9 ± 5.7^a,b^
Fat mass (kg)	16.3 ± 9.1^a^	17.8 ± 9.6^a^	8.8 ± 3.7^a^
Fat mass (%)	18.9 ± 8.1^a,b^	22.6 ± 10.7^a^	11.5 ± 3.8^b^

Data are expressed as mean ± standard deviation. Values not connected by same letter are significantly different at *p* < 0.05.

**TABLE 2:** Fasting plasma parameters of participants measured during normoxia, hypoxia intermittent hypoxia (IH-Fed) or continuous hypoxia (CH-Fasted and CH-Fed) studies.

	IH-fed		CH-fasted		CH-fed		
	Normoxia	Hypoxia	Normoxia	Hypoxia	Normoxia	Hypoxia	Between study effects (*p*-value)
ßOHB (mmol/L)	0.16 ± 0.03	0.17 ± 0.04	0.12 ± 0.02	0.13 ± 0.02	0.16 ± 0.05	0.13 ± 0.03*	0.011
NEFA (mmol/L)	0.36 ± 0.18	0.40 ± 0.21	0.41 ± 0.16	0.41 ± 0.12	0.46 ± 0.22	0.31 ± 0.17*	0.872
Glucose (mmol/L)	4.45 ± 0.67	4.70 ± 0.61	4.54 ± 0.41	4.53 ± 0.61	4.40 ± 0.18	4.61 ± 0.29*	0.928
Insulin (pmol/L)	13.29 ± 8.70	16.82 ± 8.99	14.48 ± 8.24	16.59 ± 15.50	5.19 ± 4.72	3.59 ± 2.54	0.011
HOMA-IR	0.39 ± 0.27	0.40 ± 0.26	0.43 ± 0.26	0.50 ± 0.50	0.15 ± 0.14	0.11 ± 0.08	0.030
Adipo-IR	4.57 ± 4.42	6.48 ± 5.90	6.17 ± 4.83	7.63 ± 8.34	2.11 ± 1.64	0.99 ± 0.82*	0.051

Data are expressed as mean ± standard deviation. * Indicates within subject effects significantly different at *p* < 0.05.

Participants provided informed consent prior to data collection and all methodologies were approved by the Research and Ethics Board of the University of Ottawa. Individuals with a medical history of asthma or other respiratory illness, hypertension, cardiovascular disease, diabetes, usual sleep duration of less than 7 h per night, habitual bedtime occurring after midnight, shift work and/or current smoking habit were excluded. Body weight was determined with a standard beam scale (HR-100, BWB-800AS; Tanita, Arlington Heights, IL, United States) and height was measured using a standard stadiometer (Perspective Enterprises, Portage, Michigan, United States). The percentage of fat mass, total fat mass and lean mass were determined using dual-energy X-ray absorptiometry (DXA) (General Electric Lunar Prodigy, Madison, Wisconsin; software version 6.10.019). Resting energy expenditure (REE) was measured by indirect calorimetry using a Vmax Encore 29 System metabolic cart (VIASYS Healthcare Inc, Yorba Linda, California, United States).

### Experimental Studies

This is a retrospective analysis of plasma samples collected in three different randomized crossover studies (Mahat et al., 2016, 2018; Mauger et al., 2019). For the IH-Fed study, it should be noted that 4 participants were added to the samples used in Mahat et al. (2016). In each study, participants performed two experimental sessions. Prior to each session, participants were counseled to sleep at least 7 h per night, refrain from any exercise, caffeine, and alcohol for at least 36 h, and to consume the same evening dinner the day before each session (lasagna of 3,220 kJ or 770 kcal; 42% from carbohydrates, 28% from fat, and 30% from protein).

**FIGURE 1:**
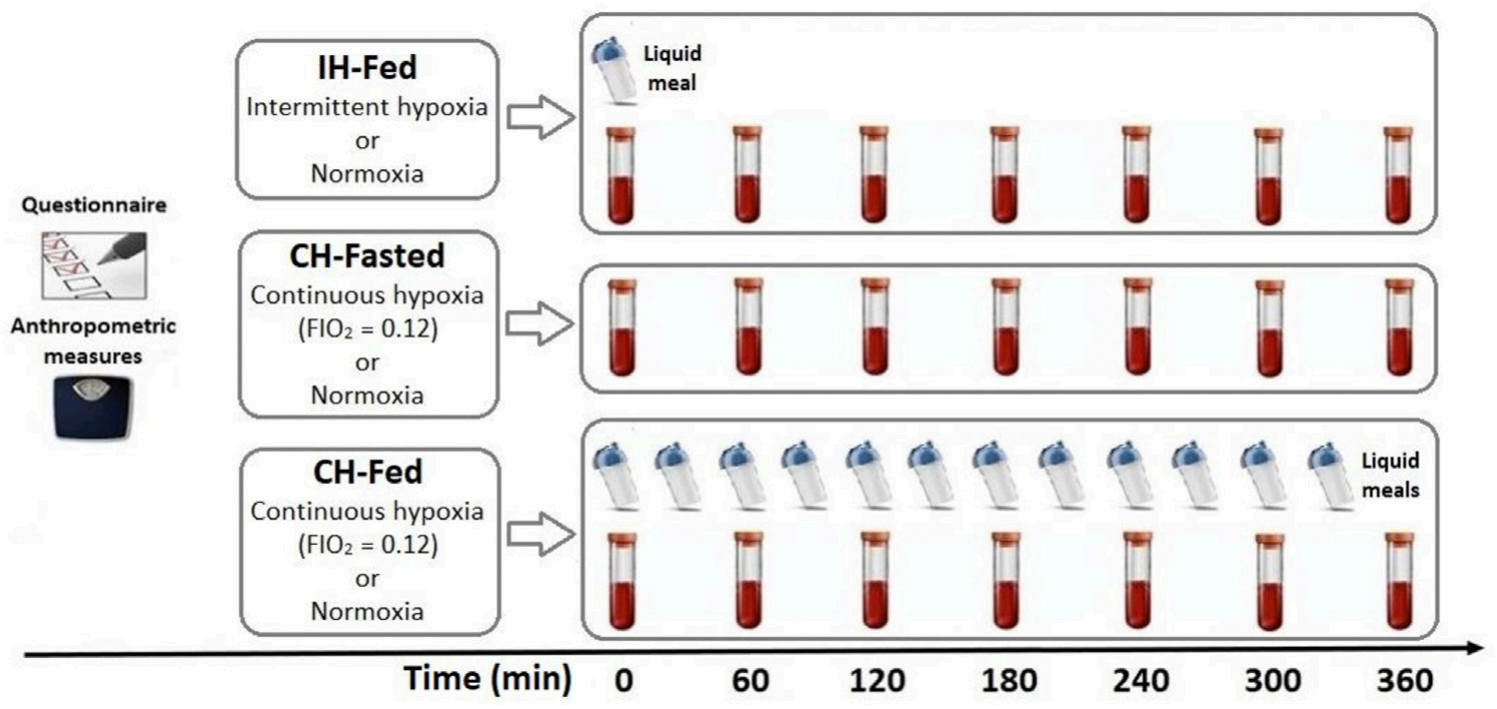
Schematic summary of the three randomized crossover studies in which ßOHB concentrations were assessed at the time indicated.In IH-Fed study, 14 healthy men were exposed to 6 h of normoxia or intermittent hypoxia (15 hypoxic events/hour) following an isocaloric meal. In the CH-Fasted study, 10 healthy men were exposed to 6 h of continuous normobaric hypoxia (FiO2 = 0.12) or normoxia in the fasting state. In the CH-fed study (CH-Fed), 9 healthy men were exposed to 6 h of normoxia or CH in aconstant Prandial state.

Each study consisted of two different sessions of 6 h: a hypoxic session and an ambient air session (Figure 1). Volunteers remained in a semi-recumbent position for the duration of the experimental session and occupied themselves by watching television. Sleep was not allowed. Oxyhemoglobin saturation and heart rates were continuously monitored by pulsed oximetry using a Masimo, Radical 7 unit (Masimo, Irvine, CA, United States). In each study, an intravenous line was inserted in the antecubital vein for blood sampling and kept patent with a continuous infusion of 0.9% saline.

#### Intermittent Hypoxia-Fed Experimental Study

On study days, volunteers arrived at the laboratory at 7:30 a.m. after a 12-h overnight fast. Volunteers were thereafter asked to consume a fat-rich liquid meal (59% of calories from fat, 28% from carbohydrates and 13% from protein), providing one-third of their estimated daily energy expenditure times a physical activity factor of 1.375 (Harris and Benedict, 1918). Participants were wearing an oro-nasal mask with a two-way Hans Rudolph non-rebreathing valve. During normoxia session, ambient air only was provided through the mask. During Intermittent Hypoxia-Fed (IH-Fed) sessions, pressurized medical N_2_ was intermittently administered until oxyhemoglobin saturation (SpO2) dropped to 85%. At this point, the flow of N_2_ was stopped and ambient air was re-administered until SpO_2_ returned to the patient’s normal value (between 95% and 100%). Intermittent hypoxia was well-tolerated and presented no adverse effects. This experimental setup allowed to produce 17.3 ± 3.8 hypoxic events per hour, which is comparable to moderate OSA.

#### Continuous Hypoxia-Fasted Experimental Study

All sessions were performed in an environmental chamber at the University of Ottawa. Volunteers arrived at the laboratory at 7:30 a.m. after a 12-h overnight fast and remained fasted for the duration of each experimental session. Drinking water was allowed. During normoxia, only ambient air was used (FIO_2_ = 0.21). During hypoxia, O_2_ extractors (CAT 12; Altitude Control Technologies, Lafayette, Colo., United States) connected to the environmental chamber kept the FIO_2_ level stable at 12%. The CAT system uses 2 stable zirconium O_2_ sensors in parallel to detect random sensors drift. The sensors are calibrated with ambient air (assuming an ambient air O_2_ concentration of 20.94%) when sensors disagree by more than 0.5% O_2_. During hypoxia, O_2_ concentration was also continuously monitored by the constantly self-calibrating Vmax system used for indirect calorimetry. O_2_ readings from both systems were always within 0.5%. To ensure the participants thermal comfort, temperature and relative humidity were stable at 28°C and 45%, respectively, and a mechanical fan was used if needed.

#### Continuous Hypoxia-Fed Experimental Study

As for Continuous Hypoxia-Fasted (CH-Fasted), all sessions were performed in an environmental chamber at the University of Ottawa. Volunteers arrived at the laboratory at 7:30 a.m. after a 12-h overnight fast. Drinking water was allowed. Volunteers were thereafter asked to consume the first of twelve liquid meals (35% of calories from fat, 55% from carbohydrates, and 10% from protein), providing a total of 40% of their estimated daily energy expenditure. Liquid meal servings were provided every 30 min. Participants were exposed to either hypoxia (FIO_2_ = 0.12) or ambient air (normoxia) for 6 h. During normoxia, only ambient air was used (FIO_2_ = 0.21) while during hypoxia, FIO_2_ level was kept stable at 12%. Calibration and thermal settings were the same as for CH-Fasted.

### Plasma Parameters

Blood samples were collected hourly, in tubes containing EDTA. Immediately after collection, plasma was obtained by centrifugation at 3,000 rpm, for 10–12 min, at 4°C. Plasma samples were kept frozen at −80°C until further analyses. Commercially available colorimetric enzymatic assays were used to measure plasma total NEFA (Wako Chemicals USA Inc, VA, United States). Insulin was measured by enzyme-linked immunosorbent assay kits (EMD Millipore, MA, United States), from which the Adipo-IR was calculated, as previously reported, by multiplying the NEFA concentration (mmol/L) by the insulin concentration (pmol/L) (Gastaldelli et al., 2017; Søndergaard et al., 2017). Adipo-IR has been shown to be a reliable and reproducible index of adipose tissue insulin resistance in both fasting and postprandial conditions in individuals with normal glucose tolerance (Gastaldelli et al., 2017). ßOHB concentration was measured using a commercial enzymatic colorimetric assay kit (Cayman Chemical, Ann Arbor, Mich., United States). Assay analyses were completed in duplicate and the intra-assay coefficients of variation were approximately <5%.

### Statistical Analysis

**FIGURE 2:**
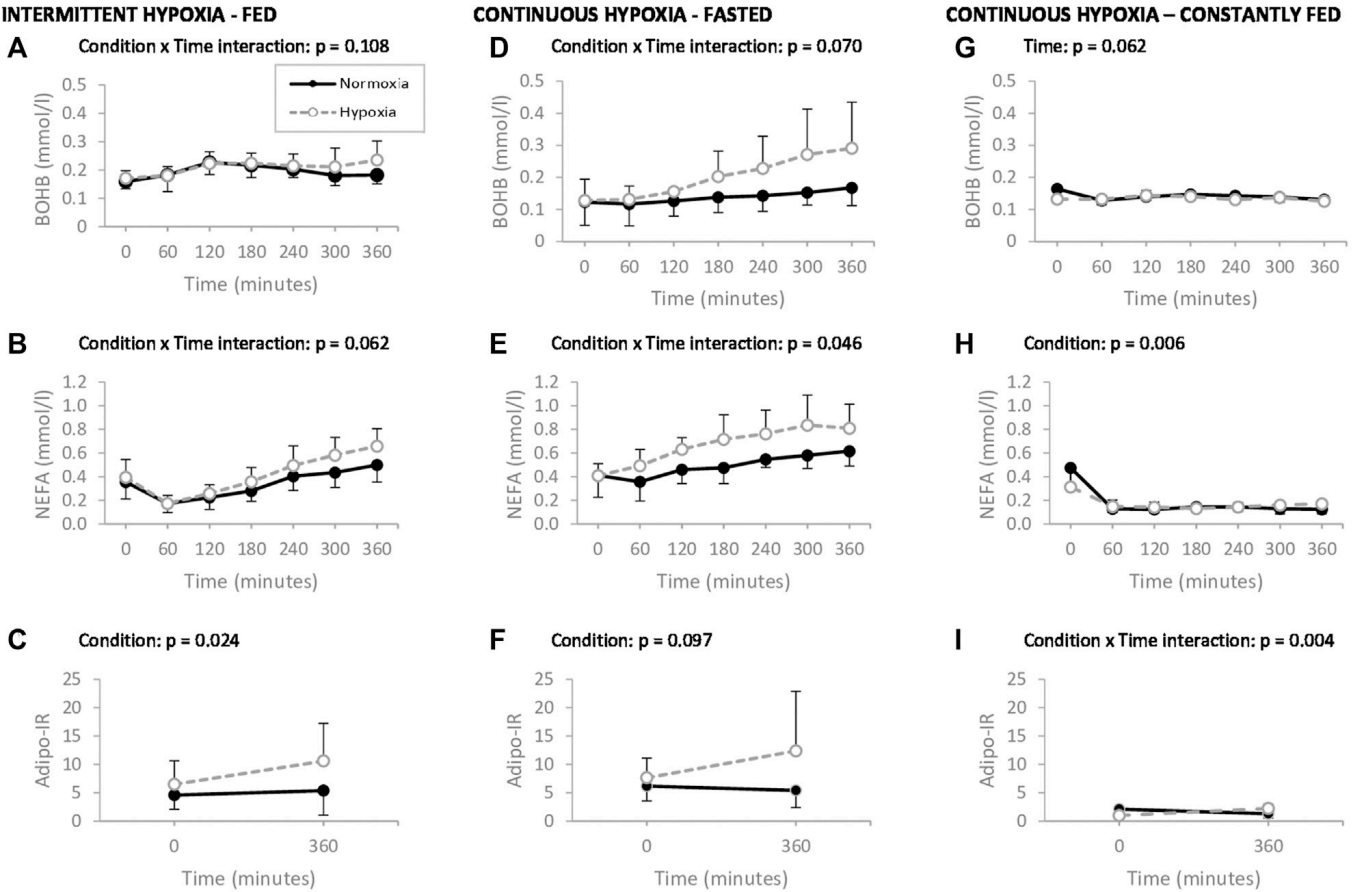
Plasma ß-hydroxybutyrate (ßOHB), nonesterified fatty acids (NEFA) and adipose tissue insulin resistance index (Adipo-IR), measured during 6 h of normoxia (–.–) or intermittent hypoxia (--o--) following an isocaloric high fat meal **(A–C)**, during 6 h of normoxia (–.–) or continuous hypoxia (--o--) under faster stat **(D–F)** and during 6 h of normoxia (–.–) or continuous hypoxia (--o--) in a constantly fed stat **(G–I)**, in healthy men. *p* values exclude time 0 in IH-Fed and CH-Fed trials. Values are means ± standard error.

All data distributions have been tested for normality. Overall, the skewness and kurtosis values fell between normal ranges, i.e., ±2 for skewness and ±7 for kurtosis (Kline, 2016). A one-way ANOVA with Tukey’s adjustment was used to compare participants’ anthropometric characteristics between studies. Fasting metabolic parameters were analyzed with repeated measures analysis of variance (ANOVA) with study as a between-subject’s parameter, and ‘normoxia’ and ‘hypoxia’ as within-subject’s parameters. For the metabolic parameters measured over time in each study, repeated measures ANOVA were performed with condition and time as within-subject’s parameters. Identification of significant interactions led to further analysis of simple main effects for hypoxia. The Greenhouse-Geisser correction was used whenever the sphericity assumption was violated. Partial eta squared are provided as an estimate of effect size. For IH-Fed and CH-Fed, time 0, which corresponds to fasting state, were excluded to account for prandial and postprandial effects only. Error bars in Figures 2A–I were adjusted to eliminate between subjects’ variability and better reflect the statistical power of the study crossover design (Cousineau et al., 2005). A level of significance of *p* < 0.05 was considered statistically significant. Jamovi version 1.2.27.0 for Windows was used for data analysis (The Jamovi project, Sydney, NSW, Australia).

## Results

### Characteristics of Participants

As reported in Table 1, no statistical differences were observed between participants of each study with exception of mean lean mass, which was significantly higher in IH-Fed vs. CH-Fasted (*p* = 0.043), as well as mean fat mass %, which was significantly lower in CH-Fed vs. Ch-Fasted (*p* = 0.015). Mean fat mass tended to be lower in CH-Fed vs. CH-Fasted (*p* = 0.059).

Fasting plasma parameters are reported in Table 2. Fasting ßOHB levels differed between IH-Fed and CH-Fasted while both studies presented no significant difference in ßOHB fasting levels of CH-Fed study (*p* = 0.011). Fasting insulin levels for the CH-Fed study were significantly lower than fasting levels in IH-Fed and CH-Fasted (*p* = 0.011). Fasting HOMA-IR (Homeostatic Model Assessment of Insulin Resistance) and Adipo-IR levels differed between CH-Fed and CH-Fasted while both studies presented no significant difference for the same parameters in IH-Fed (*p* = 0.030 and *p* = 0.051 respectively). There were significant differences in ßOHB, insulin, HOMA-IR and Adipo-IR between normoxia and hypoxia in CH-Fed.

### Effects of Hypoxia on Plasma Parameters

Plasma ßOHB, NEFA, and Adipo-IR levels during 6 h of normoxia or hypoxia are shown in Figures 2A–I.

In the IH-Fed study, which was conducted in the postprandial state, ßOHB levels (Figure 2A) tended to evolve in a different manner over time between normoxia and hypoxia (condition × time interaction, *p* = 0.108, η_p_^2^ = 0.171). More specifically, ßOHB levels transiently increased after the meal and returned to initial levels after 6 h in normoxia but remained elevated during hypoxia. Average ßOHB concentrations were increased by 34% compared to fasting levels after 6 h of IH-Fed whereas a marginal increase of only 3% was observed under normoxia. A trend toward greater increase in plasma NEFA levels over time was observed under IH-Fed compared to normoxia (condition × time interaction, *p* = 0.062, η_p_^2^ = 0.168) (Figure 2B). The Adipo-IR was significantly higher under IH-Fed than normoxia regardless of time (main effect of condition, *p* = 0.024, η_p_^2^ = 0.333) (Figure 2C). Irrespective of experimental conditions, the Adipo-IR increases over 360 min of exposure, but this fell short of statistical significance (effect of time, *p* = 0.090, η_p_^2^ = 0.206).

In the CH-Fasted study, ßOHB levels increased over time in both experimental conditions and this increase tended to be greater under CH-Fasted (condition × time interaction, *p* = 0.070, η_p_^2^ = 0.307) (Figure 2D). The mean average increase in ßOHB was of 133% and 38% by the end of the 6-hour session under CH-Fasted and normoxia, respectively. Similarly, NEFA concentrations significantly increased over 6 h in both experimental conditions with a greater increase observed under continuous hypoxia (condition × time interaction, *p* = 0.046, η_p_^2^ = 0.254) (Figure 2E). The Adipo-IR tended to be greater under CH-Fasted than under normoxia, regardless of time (main effect of condition, *p* = 0.097, η_p_^2^ = 0.276) (Figure 2F).

In the CH-Fed study, ßOHB levels slightly increased up to 180 min before falling back to initial concentrations by the end of the protocol in both normoxia and CH (main effect of time, *p* = 0.062, η_p_^2^ = 0.294) (Figure 2G). Mean plasma NEFA levels were significantly higher under CH than normoxia, regardless of time (main effect of condition, *p* = 0.006, η_p_^2^ = 0.633) (Figure 2H). Adipo-IR significantly increased over time in hypoxic conditions while it decreased under normoxia (condition × time interaction, *p* = 0.004, η_p_^2^ = 0.671) (Figure 2I).

## Discussion

This study evaluated the hypothesis that acute normobaric hypoxia exposure, which elevates circulating NEFA levels, would augment plasma ßOHB concentrations. We corroborated that acute normobaric hypoxia, conducted in controlled environment, elevates circulating NEFA levels and we confirmed the hypothesis that this is accompanied by an increase in plasma ßOHB concentrations in healthy men. The hypoxic ßOHB rise is however repressed by prandial and postprandial status. We also determined that upon hypoxic exposure, Adipo-IR, a surrogate of adipose tissue resistance to insulin, is increased.

To our knowledge, this is the first study examining the impact of acute hypoxic exposure on ketonemia in humans. Using different modalities of normobaric hypoxia exposure (intermittent or continuous) and feeding status (fasting, postprandial or prandial), we report that hypoxia, either intermittent or continuous, tends to elicit greater elevations in ßOHB levels during postprandial state (IH-Fed) and fasting state (CH-Fasted). The increased ketogenic response observed upon hypoxia is however abrogated for the first two to three postprandial hours (IH-Fed) and under a constantly fed state (CH-Fed). These results are in line with previous observations in rodent studies showing that hypoxia increases blood ketone levels. Indeed, in a series of experiments aiming at understanding the physiological responses associated with survival time under hypoxia, Rising *et al.* (Rising & D’Alecy, 1989) reported a significant 267% increase in ßOHB levels 30 min following short exposures (90–150 s) to severe hypoxic conditions (FiO_2_ = 0.046). The same group also reported dose-dependent increases in ßOHB levels in rats and ground squirrels following 5, 10 and 60 min exposures to hypoxic conditions (FiO_2_ = 0.045) (D’Alecy et al., 1990). Ground squirrel showed greater increases in blood ßOHB than rats (517% vs. 378%, respectively) after the 60 min exposure, which suggests important inter-species differences in ketone metabolism adaptation to hypoxia. More recently, Jun *et al.* (2012) reported a significant 3-fold increase in ketonemia in mice exposed for 6 h at FiO_2_ = 0.07 while no changes in ketonemia were noted in less severe hypoxic conditions (FiO_2_ = 0.10, 0.14 and 0.17). Together, these observations and ours demonstrate that hypoxia leads to an increase in ketonemia.

The physiological mechanisms responsible for the increase in ketonemia under hypoxic conditions in rodents and in humans have yet to be elucidated. In that respect, we suggest that the observed increase in ketonemia is the result of a rate of ketone production exceeding the rate of ketone utilization. This assumption rests on several studies of Balasse et Féry (Balasse and Féry, 1989), who demonstrated, with tracer infusions, that the maximal disposal rate of KB is around 2.5 mmol/min in humans when KB concentration is above 10 mM, a concentration way beyond what our participants reached. Yet, whether acute hypoxia may further limit the uptake and utilization of KB in humans remains unknown. In that regard, several physiological responses to hypoxia are likely to disturb the balance between ketone production and utilization. First, there is the increase in sympathetic tone in response to hypoxia (Somers et al., 1989; Oltmanns et al., 2004), which is well recognized to stimulate adipose tissue lipolysis (Rayner, 2001) and thus, lead to a rise in NEFA levels, as observed in the current and other human studies (Jun et al., 2011; Mahat et al., 2016, 2018; Chopra et al., 2017; Mauger et al., 2019; Morin et al., 2021). Since plasma NEFA are the main substrate for ketogenesis (Robinson and Williamson 1980), the hypoxia-induced increase in sympathetic tone and its stimulating effect on NEFA levels is likely to stimulate ketogenesis in the liver. While our experiments did not include direct measurement of sympathetic tone, our hypoxia experimental sessions, especially the ones under continuous hypoxia, were reported to significantly increase heart rate by 20% (Mahat et al., 2018; Mauger et al., 2019), which would most likely reflect a sympathetic activation.

The reduction of adipose tissue insulin sensitivity observed in response to hypoxia in the current study, as estimated by the Adipo-IR index, could also favor the release of NEFA into circulation by attenuating the insulin-suppressive effect on adipose tissue lipolysis (Lafontan and Langin, 2009; Young and Zechner, 2013). It is interesting to note that when systemic insulin sensitivity surrogate indexes such as the HOMA-IR (Matthews et al., 1985) and the Matsuda index (Matsuda and DeFronzo, 1999), which are based on fasting or postprandial plasma insulin and glucose concentrations, no change of insulin sensitivity was observed in response to hypoxia (data not shown). This latter observation is not in line with other studies in humans which reported that insulin sensitivity is attenuated in response to continuous (Peltonen et al., 2012) or intermittent hypoxia (Louis and Punjabi, 2009) protocols comparable to the ones we conducted. However, the methods used for quantifying insulin sensitivity, e.g., hypersinsulinemic euglycemic clamp and intravenous glucose tolerance test, in these previous studies were more direct, which likely explains the divergence with our insulin sensitivity surrogate indexes. Nonetheless, the fact that hypoxia significantly affected Adipo-IR index in the current study could be interpreted as an indication that adipose tissue insulin sensitivity may precede systemic insulin sensitivity in response to hypoxia. Such a hypothesis however needs to be formally tested.

An alternate hypothesis to explain the increase in ketonemia upon hypoxic exposure involves hepatocyte metabolism. Previous *in vitro* studies showed that hypoxia favors fatty acids uptake (Hazlehurst et al., 2022) and impairs fatty acid oxidation in hepatocytes (Cao et al., 2014; Liu et al., 2014), which, in turn, could favor the channelling of acetyl-CoA into the ketogenic pathway under hypoxia.

Of note, we report an absence of difference in ßOHB levels between normoxia and hypoxia during the first 3 hours of postprandial (IH-Fed) and prandial (CH-Fed) states, suggesting ketonemia is mostly driven by the meal. We also observed an increase in ketonemia during the first hours of the postprandial and prandial states despite that this period corresponded to a significant reduction in circulating NEFA (Figures 2B,H). This is intriguing in view that NEFA are the main precursors for ketogenesis. Nonetheless, these results corroborate with the findings of Halkes et al. (2003) and Meijssen et al. (2000), who both demonstrated a rise in ketonemia following an oral fat-loading test. This potential increased conversion of NEFA-derived acetyl-CoA into ßOHB, which seems influenced by the lipid content of the meal, may be directed toward lowering the surge of dietary NEFA and/or maintaining a minimal level of ßOHB to sustain ketogenic functions.

There were anthropometric differences between our participants, notably in fat mass %. Despite the observed difference in body fat %, participants’ mean body fat % in each study fit in the ‘good and fair’ categories, based on Fitness Categories for Body Composition (% Body Fat) for Men by Age (American College of Sports Medicine et al., 2014). Although fat mass and fat mass % may influence ketonemia (Nosadini et al., 1985; Inokuchi et al., 1992), the precise relationship between fat mass % and ßOHB plasma concentration is not yet established. It is unknown whether leaner individuals would produce more ßOHB under hypoxic conditions in comparison to individuals with higher adiposity. However, it is possible to speculate that healthy individuals with normal adiposity would have a stronger ketogenic response to hypoxia, when compared to healthy individuals with obesity, but ßOHB uptake may also be increased, thus making the elaboration of a hypothesis even more difficult/complex. We believe the adipose tissue insulin resistance is a stronger predictor of ketonemia than fat mass percentage, as insulin sensitivity limits adipose tissue lipolysis, which decreases the amount of NEFA available for ketogenesis. Nonetheless, it would be interesting to investigate ßOHB production rate in individuals of different fat mass % to better understand the relationship between said variables.

Some limitations and strengths of this study warrant discussion. First, only plasma ßOHB concentrations were measured which forbids us from inferring about a possible increase in ketogenesis. Additionally, our samples consisted solely of healthy young men, which prevents us from extending our conclusions to women, older adults, and less healthy individuals. The main strength of the present study lies on the statistical strength of the crossover design, that counterbalance the relatively small sample size of each study so that changes in plasma levels of ßOHB were detected. The comparison of three different prandial status additionally highlights the influence of both fasted and fed status over ketonemia under acute hypoxia.

## Conclusion

We tested whether acute normobaric hypoxia would raise circulating ßOHB levels in response to the anticipated elevations in NEFA levels. Although no direct link or mechanism can be inferred from our observations, we found that acute normobaric hypoxia tends to elevate circulating NEFA and ßOHB concentrations in healthy men. The ketonemic effect of hypoxia is however abrogated in the hours following the ingestion of a meal while the effect upon NEFA levels is considerably reduced. Ketone bodies are important metabolic and signaling mediators (Newman and Verdin, 2014b; Puchalska and Crawford, 2017). Hence, further understanding of the regulatory and metabolic cascade leading to changes in ketone bodies production can provide further insights into the homeostatic responses of humans to oxygen deprivation.

## Data Availability

The raw data supporting the conclusions of this article will be made available by the authors, without undue reservation.
